# Supplementary material for: Prevalence and correlates of pregnancy self-testing among pregnant women attending antenatal care in western Kenya
Source: PLoS One. 2021 Nov 12;16(11):e0258578. doi: 10.1371/journal.pone.0258578 (PMC8589148; doi:10.1371/journal.pone.0258578)
Supplement: S1 Appendix — (PDF) [file pone.0258578.s001.pdf]

# Demographics

| | |
|---|---|
| Participant ID | ______________________ |
| CRF Version number | ◯ 2.0 |
| Purpose: This form is used to capture demographic information about the study participant | |
| Script: Thank you for joining this study. The following survey will ask you questions about your life, your beliefs, and your behavior. Some of the questions we ask about your behavior you may consider to be private or confidential. We are asking these questions because your answers should help us to understand whether PrEP could help slow the spread of HIV in your community. The information you provide is an important contribution to this study and will be kept confidential. You can skip any question that makes you feel uncomfortable or stop taking the survey at any time. Some questions will ask you about your behavior during a specific time period. Please pay close attention to the time period and only tell us about your behavior during that time. | |
| Has client completed consent? | ◯ Yes ◯ No<br>(If no, do not complete any further questions and ensure client completes consent before proceeding with enrollment CRFs) |
| Do you know your date of birth? | ◯ Yes ◯ No |
| What is your date of birth? | ______________________ |
| What is your age? | ______________________ |
| Sex | ◯ Male<br>◯ Female |
| What is your marital status? (check one) | ◯ Currently married ◯ Divorced/separated<br>◯ Come we stay ◯ Steady boyfriend<br>◯ Never married ◯ Widow<br>◯ Single ◯ Prefer not to answer |
| Is your marriage: | ◯ Monogamous ◯ Polygamous<br>◯ Prefer not to answer |
| Are you currently in school? | ◯ Yes ◯ No |
| Number of complete years in school | ______________________ |
| Do you have regular employment? | ◯ Yes ◯ No ◯ Prefer not to answer |
| How long does it take you to travel to clinic? : # of minutes | ______________________ |
| How many people usually reside in your house (including yourself)? | ______________________ |

# Pregnancy History

| | |
|---|---|
| Participant ID | __________________________________ |
| CRF Version | ◯ 2.0 |
| Purpose: This form is used to collect information about each participants obstetric history | |
| Script: I will now ask you a few questions about your health and any previous pregnancies you may have had. | |
| Have you been pregnant before? | ◯ Yes ◯ No |
| Total number of pregnancies (include current pregnancy if applicable)? | __________________________________ |
| Total number of live births? | __________________________________ |
| Total number of living children? | __________________________________ |
| Total number of miscarriages, including spontaneous abortions and induced abortions? | __________________________________ |
| Total number of still births? | __________________________________ |
| Do you know the date your last pregnancy ended? | ◯ Yes ◯ No |
| What date did your last pregnancy end? | __________________________________ |
| If unknown, what is the age of your youngest child? | __________________________________ |
| Did your last pregnancy end in a live birth? | ◯ Yes ◯ No |
| Location of last delivery? | ◯ Health care facility ◯ Home<br>◯ Friend or relative home<br>◯ Other ◯ Not applicable |
| If other, specify | __________________________________ |

| | |
|---|---|
| For your last delivery, what were the reasons for delivering where you did (tick all that apply)? | ☐ Medical expertise ☐ Cost/money ☐ Distance/convenience of location ☐ Transport ☐ Previous experience ☐ Safety ☐ Fear of theft/misplacement of baby ☐ Family/culture/tradition ☐ Recommendation or advice of friends/family ☐ Obstetric or gynecologic complication ☐ Other ☐ No answer |
| Mode of last delivery (if applicable): | ◯ Vaginal ◯ C-section ◯ No answer |
| Any history of c-section? | ◯ Yes ◯ No |
| Did you have problems with your last pregnancy (tick all that apply)? | ☐ No problem ☐ Late pregnancy bleeding ☐ Miscarriage ☐ Fetal miscarriage ☐ High blood pressure ☐ Other |
| If other, specify | ____________________________ |
| Do you have a history of any of the following? | ☐ No Problem<br>☐ Laceration<br>☐ Infection<br>☐ Hemorrhage<br>☐ Obstructed delivery<br>☐ Premature Birth (< 37 weeks)<br>☐ Spontaneous fetal death and/or stillbirth (< 20 weeks)<br>☐ Other |
| Did you have problems with your last delivery (tick all that apply)? | ☐ No Problem ☐ Laceration ☐ Infection ☐ Hemorrhage ☐ Obstructed delivery ☐ Premature Birth (< 37 weeks) ☐ Spontaneous fetal death and/or stillbirth (< 20 weeks) ☐ Other |
| If other, specify | ____________________________ |
| Is current gestational age known? | ◯ Yes ◯ No |
| If gestational age is unknown, is participant presumed to be ? 36 weeks gestation? | ◯ Yes ◯ No |
| Current Gestational age: Weeks | ____________________________ |
| Is the date of last menstrual period known? | ◯ Yes ◯ No |
| Date of last menstrual period: | ____________________________ |

| | |
|---|---|
| How did you obtain the date of the last menstrual period? | ◯ Abstracted from MCH card<br>◯ Self-reported<br>◯ Self report and confirmed on MCH card |
| How certain about calendar month for last menstrual period? | ◯ certain ◯ uncertain |
| What is the fundal height? | ______________________ |
| Estimated date of delivery: | ______________________ |
| How did you initially suspect that you were pregnant? | ___________________________ |
| Once you suspected that you were pregnant, how did you confirm that you were pregnant? | ◯ Conducted a pregnancy test yourself<br>◯ Went to a public health care facility<br>◯ Went to a private health care facility<br>◯ Other<br>◯ |
| Other, specify | ______________________ |
| For users of pregnancy tests: Where did you obtain the pregnancy test? | ◯ Chemist<br>◯ Public Health Care Facility<br>◯ Private Health Care Facility<br>◯ Store<br>◯ Other<br>◯ |
| Other, specify | ______________________ |
| For non-users of pregnancy tests: Why did you not use a pregnancy test? | ◯ Did not know I could use a pregnancy test<br>◯ Did not have the money to pay for a pregnancy test<br>◯ Did not think it was necessary<br>◯ Other<br>◯ |
| Other, specify | ______________________ |
| When did you confirm your pregnancy? | ◯ First Trimester (0-13 weeks)<br>◯ Second Trimester (14-26 weeks)<br>◯ Third Trimester (27-40 Weeks)<br>◯ No answer<br>◯ |
| When did you first present for antenatal care? | ◯ First Trimester (0-13 weeks)<br>◯ Second Trimester (14-26 weeks)<br>◯ Third Trimester (27-40 Weeks)<br>◯ |

| | |
|---|---|
| Diabetes | ◯ Yes ◯ No ◯ unknown<br>(MCH Card Page 5) |
| Hypertension | ◯ Yes ◯ No ◯ unknown<br>(MCH Card Page 5) |
| Tuberculosis | ◯ Yes ◯ No ◯ unknown<br>(MCH Card Page 5) |
| Hepatitis b | ◯ Yes ◯ No ◯ unknown<br>(MCH Card Page 7) |
| Serology (VDRL/RPR) | ◯ Reactive ◯ Non-reactive<br>◯ unknown ◯ Not Done<br>(MCH Card Page 7) |
| TB Screening | ◯ Yes ◯ No ◯ unknown<br>(MCH Card Page 7) |
| HIV | ◯ Reactive<br>◯ Non-Reactive<br>◯ Not Tested<br>(MCH Card Page 7) |
| Number of ANC Visits, including current visit | ______________________<br>(MCH Card Page 8) |
